# Development of event-specific detection method for identification of insect resistant NIBGE-1601 cotton harboring double gene Cry1Ac-Cry2Ab construct

**DOI:** 10.1038/s41598-021-82798-w

**Published:** 2021-02-10

**Authors:** Muhammad Asif, Hamid Anees Siddiqui, Rubab Zahra Naqvi, Imran Amin, Shaheen Asad, Zahid Mukhtar, Aftab Bashir, Shahid Mansoor

**Affiliations:** 1 Agricultural Biotechnology Division, National Institute for Biotechnology and Genetic, Engineering, Faisalabad, Pakistan; 2 Pakistan Institute of Engineering and Applied Sciences, Nilore, Pakistan; 3 Department of Biological Sciences, Forman Christian College, Lahore, Pakistan

**Keywords:** Biotechnology, Molecular biology, Plant sciences

## Abstract

Bt cotton expressing Cry1Ac is being cultivated in Pakistan. It has been observed that pink bollworm may have developed resistance against single Bt gene (Cry1Ac). For durable resistance, insect resistant NIBGE-1601 cotton harboring double gene Cry1Ac-Cry2Ab construct was developed. There was a need to characterize NIBGE-1601 event for intellectual property rights protection. The Presence of NIBGE Cry1Ac and NIBGE Cry2Ab genes was checked in NIBGE-1601 cotton plants through PCR, while there was no amplification using primers specific for Monsanto events (MON531, MON15985, MON1445). Using genome walking technology, NIBGE-601 event has been characterized. Event-specific primers of NIBGE-1601 were designed and evaluated to differentiate it from other cotton events mentioned above. NIBGE-1601 event detection primers are highly specific, therefore, can detect NIBGE 1601 event at different conditions using single or multiplex PCR. In the qualitative PCR, using NIBGE-1601 event specific primers, 0.05 ng was the limit of detection for NIBGE-1601double gene cotton genomic DNA. Thus event characterization and development of event-specific diagnostics will help in breeding new cotton varieties resistant to cotton bollworms.

## Introduction

Genetically modified (GM) crops have been developed in many countries of the world for tolerance to biotic and abiotic stresses. During 2017, GM crops have been planted on 190 million hectares in 24 countries^1^. Cartagena Protocol on Biosafety governs trans-border movement of genetically modified organisms (GMOs) at international level and permits governments to ban importation of any GMO where there are biosafety concerns. Pakistan has approved National Biosafety Guidelines and Pakistan Biosafety Rules 2005 for safe handling of GMOs at all levels^2^. There is a strict process of biosafety assessment before the commercial release of a GM crop and development of transgenic event-specific transgenic diagnostics helps in the detection of event whose biosafety has been accomplished. The detection of GMOs has become necessary to comply with labeling regulations because several countries have imposed threshold levels for labeling GM products to meet the demand of traceability and consumers right to know about the product^3^. International regulations make it imperative for the governments, food or feed producers and diagnostic labs to develop reliable methods for detection of GMOs.

Genetically modified crops (GMCs) can be commercialized if found safe after thorough biosafety studies. Characterization of GMCs is a critical component of risk assessment because precise knowledge of transgene localization in the genome, copies of inserts and genomic sequences flanking transgene cassettes is essential for biosafety assessment of GMCs. It also helps in establishment of validated methods for reliable detection of transgenics to meet the requirements of labeling regulations, traceability and monitoring. Event characterization is also an essential requirement for protection of a developer’s intellectual property rights (IPRs). Therefore, molecular characterization of transgenics is of particular importance for all the stakeholders including producers, risk assessors, regulators and consumers of GM crops^4–6^.

GMO detection protocols are generally based on protein and DNA assays^7^.

Protein-based GMO tests are western blot and immuno-assays (ELISA and lateral flow strips), while DNA-based GMO detection includes Southern blot and polymerase chain reaction (qualitative and quantitative PCR). PCR achieves the greatest sensitivity and specificity to meet regulatory requirements^8^. Molecular characterization of transformation events is mostly performed by PCR amplification and Southern hybridization^9^. Transgenic events are characterized by identifying the unknown DNA sequences flanking the transgene cassettes in the genome, mostly by using genome walking (GW) approaches. Several methods of GW have been developed and more recently are applied in combination with next generation sequencing (NGS) technologies^10–13^. Genome walking strategies are increasingly being employed because of the minimal requirement of expensive equipment. Three categories of GW approaches are: (i) restriction-based GW, in which genomic DNA is restricted and then ligated with adapters, (ii) primer-based GW, wherein random or degenerate primers along with specific primers are used for PCR, (iii) extension-based GW that involves extension of a sequence specific primer and then 3′-tailing of single strand DNA^11,13,14^.

Bt cotton expressing Cry1Ac (MON531 event) is being grown on a large area in Pakistan. Pink bollworm (*Pectinophora gossypiella*) has developed resistance against single Bt gene (Cry1Ac). Insect resistant transgenic cotton expressing more than one insecticidal genes can provide durable and better control of insect pests^15^. Insect resistant NIBGE-1601 cotton harboring double gene Cry1Ac-Cry2Ab construct has shown resistance against bollworms and armyworm^16^. Present research work was conducted to develop event-specific detection method for identification of NIBGE-1601 cotton. To meet regulatory requirements and properly address the IPRs, NIBGE-1601 cotton event has been successfully characterized using genome walking technology. NIBGE-1601 event specific PCR primers have been evaluated to differentiate from Monsanto and other cotton lines including non-transgenic coker-312 and triple gene NIBGE cotton event.

## Results

### PCR for transgene analysis of NIBGE-1601 cotton plants

**Figure 1:**
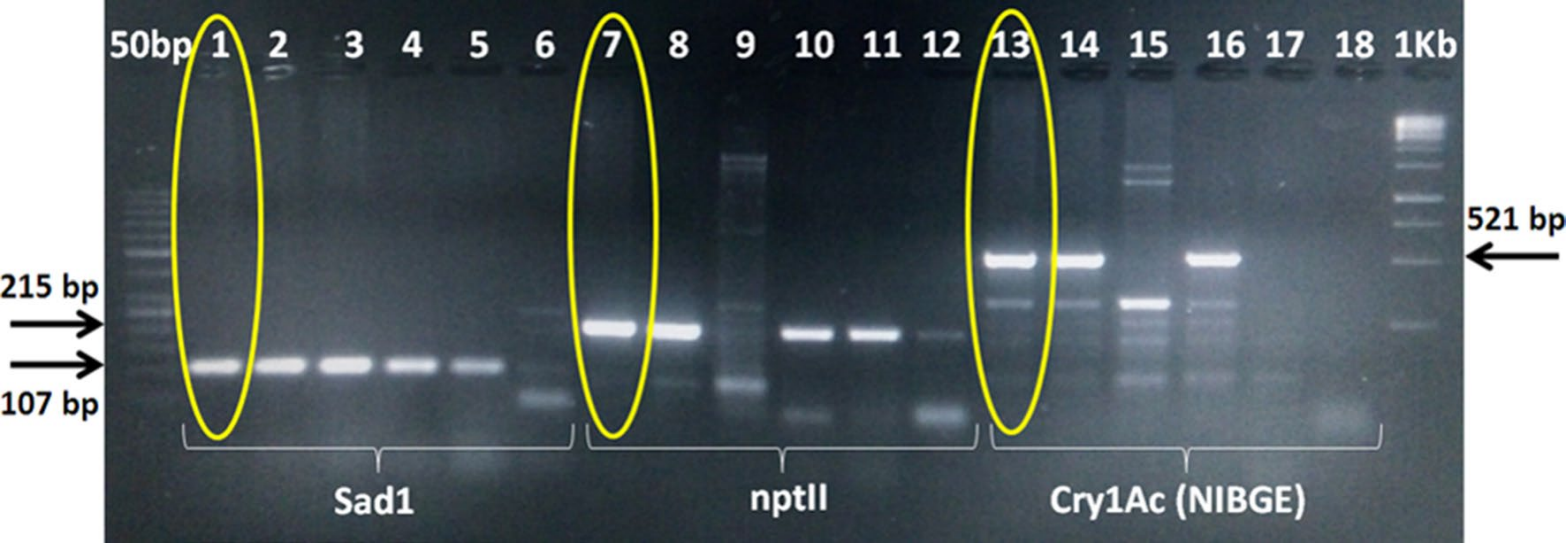
PCR for transgene analysis of NIBGE-1601 cotton event using SadI, nptII and Cry1Ac specific primers. 1 = NIBGE-1601 cotton, 2 = Triple gene (TG) NIBGE cotton (P1), 3 = non-GM Coker (P2), 4 = TG NIBGE cotton (P2), 5 = Bollgard-II, 6 = negative control (water), 7 = NIBGE-1601 cotton, 8 = TG NIBGE cotton (P1), 9 = non-GM Coker (P2), 10 = TG NIBGE cotton (P2), 11 = Bollgard-II, 12 = negative control (water), 13 = NIBGE-1601 cotton, 14 = TG NIBGE cotton (P1), 15 = non-GM Coker (P2), 16 = TG NIBGE cotton (P2), 17 = Bollgard-II, 18 = negative control (water)* Primers: S1F + S2R (Sad1; cotton fiber gene specific, 107 bp), APH2 Short + APH2 Reverse (nptII, 215 bp), CR1BDF5 + CR1BDR5 (NIBGE Cry1Ac gene specific, 521 bp).

**Figure 2:**
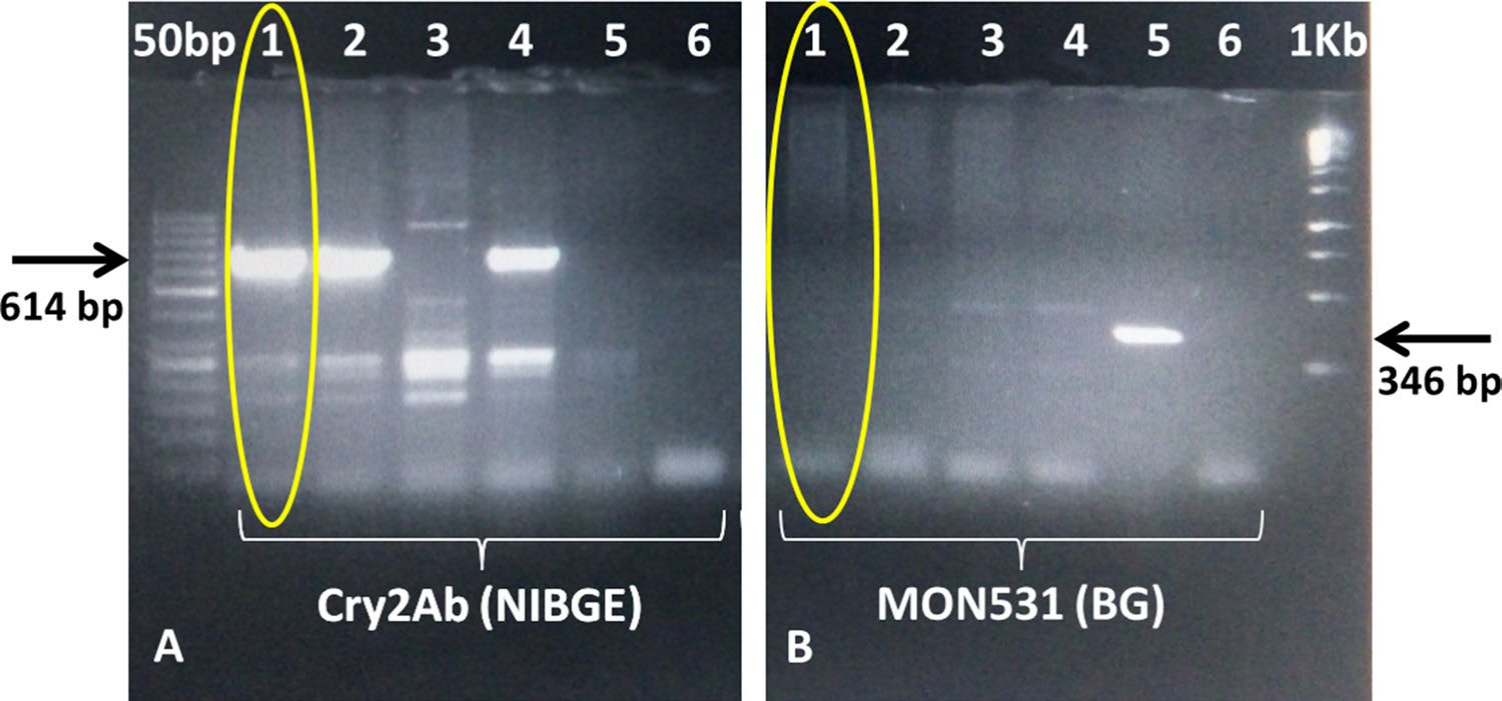
PCR for transgene analysis of NIBGE-1601 cotton event using Cry2Ab and Mon531 specific primers. Panel A): 1 = NIBGE-1601 cotton, 2 = Triple gene (TG) NIBGE cotton (P1), 3 = non-GM Coker (P2), 4 = TG NIBGE cotton (P2), 5 = Bollgard-II, 6 = negative control (water), Panel B): 1 = NIBGE-1601 cotton, 2 = TG NIBGE cotton (P1), 3 = non-GM Coker (P2), 4 = TG NIBGE cotton (P2), 5 = Bollgard-II, 6 = negative control (water) *Primers: CR2BDF4 + CR2BDR4 (NIBGE Cry2Ab gene specific,614 bp), C1F + C2R (MON531, BG event specific, 346 bp).

**Figure 3:**
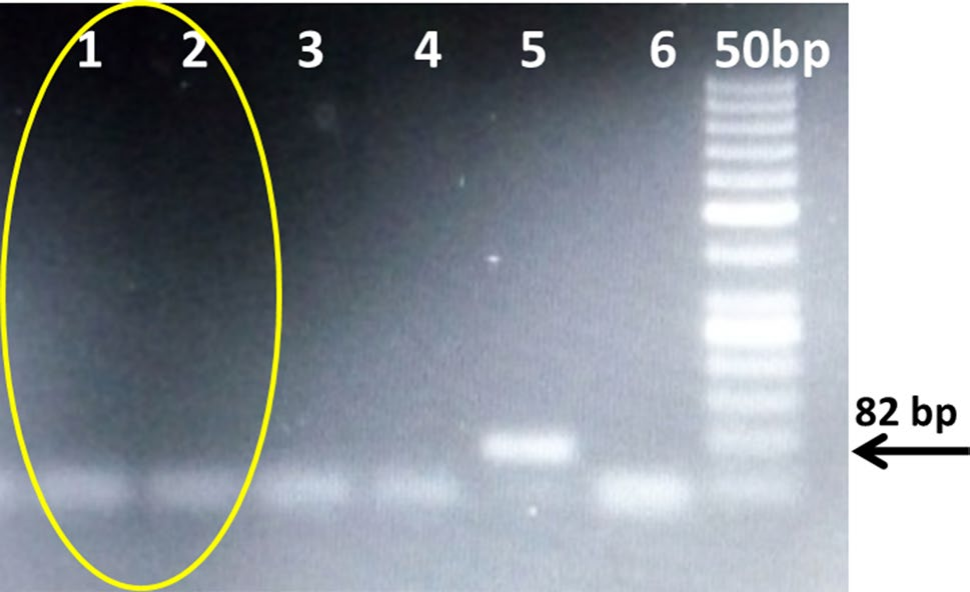
PCR for transgene analysis of NIBGE-1601 cotton event using Mon15985 specific primers. 1, 2 = NIBGE-1601 cotton plants; 3, 4 = non-GM Coker-312 (P1, P2), 5 = Bollgard-II, 6 = negative control (water) * Primers: 13_109F and 13_109R (Mon15985, BGII event specific, 82 bp).

**Figure 4:**
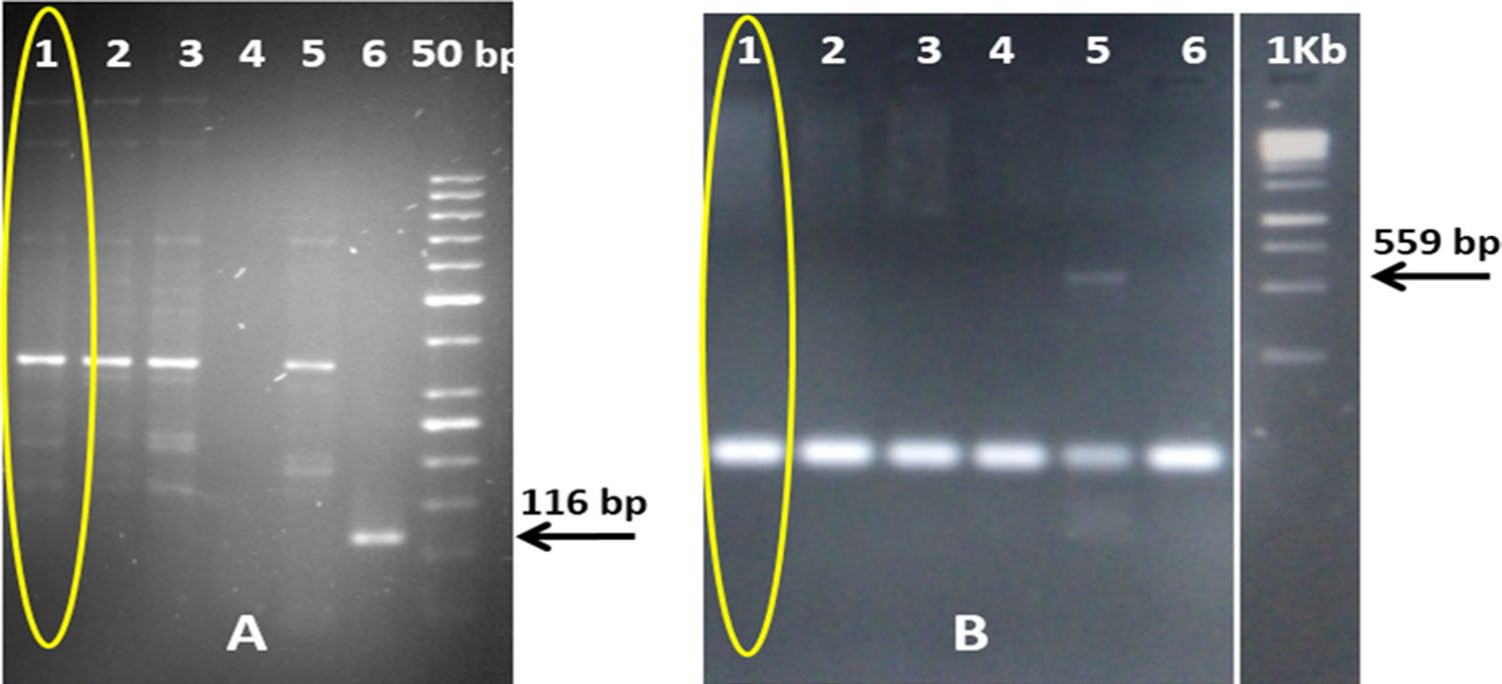
PCR for transgene analysis of NIBGE-1601 cotton event using Mon15985 and Mon1445 specific primers. Panel A): 1 = NIBGE-1601 cotton, 2 = Triple gene (TG) NIBGE cotton (P1), 3 = non-GM coker 312 (P2), 4 = negative control (water), 5 = non-GM coker 312 (P1), 6 = Bollgard-II; Panel B): 1 = NIBGE-1601 cotton, 2 = TG NIBGE cotton (P1), 3 = non-GM Coker-312 (P2), 4 = TG NIBGE cotton (P2), 5 = RR cotton, 6 = negative control (water) * Primers: A) CTCR2F + CTCR2R (Mon15985, BGII event specific, 116 bp); B) E1F + E2R (Mon1445, RR event specific, 559 bp).

PCR of T_0_ and T_1_ plants of the C2-E36-P1 (NIBGE-1601) cotton line showed the presence of selectable marker (nptII), NIBGE Cry1Ac and NIBGE Cry2Ab genes. NIBGE-1601 cotton event is different from Monsantro cotton events because Monsanto events (MON531, MON15985, MON1445) specific PCR products were not detected in the NIBGE-1601 cotton line (Table 2, Figs. 1, 2, 3, 4, Supplementary Fig. S1–S4). Positive plants of T_1_ and T_2_ progenies of NIBGE-1601 were also tested and the PCR results were similar to that of its T_0_ parent (Supplementary Fig. S6).

### Event characterization of NIBGE-1601 cotton

**Figure 5:**
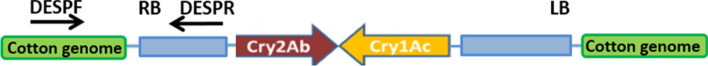
Event characterization of insect resistant NIBGE-1601 cotton.

**Figure 6:**
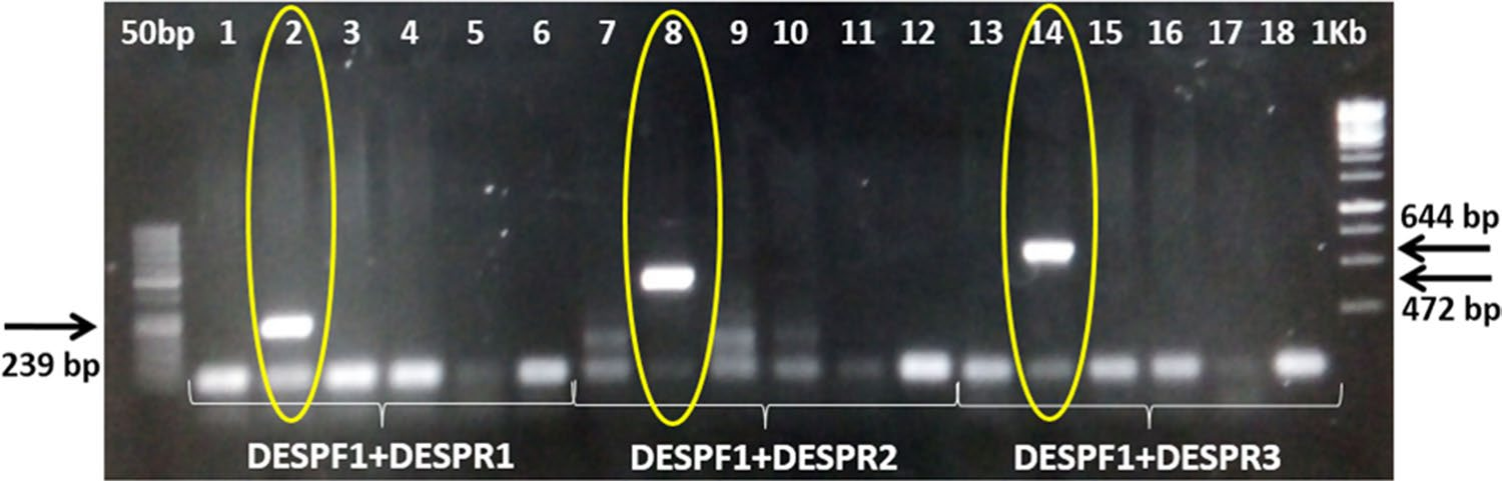
Event specific PCR of NIBGE-1601 insect resistant cotton. 1 = IR-NIBGE-3701 (MON531), 2 = NIBGE-1601 cotton, 3 = Triple gene (TG) NIBGE cotton, 4 = non-GM Coker-312 (P1), 5 = Bollgard-II, 6 = negative control (water), 7 = IR-NIBGE-3701 (MON531), 8 = NIBGE-1601 cotton, 9 = TG NIBGE cotton , 10 = non-GM Coker-312 (P1), 11 = Bollgard-II, 12 = negative control (water), 13 = IR-NIBGE-3701 (MON531), 14 = NIBGE-1601 cotton, 15 = TG NIBGE cotton , 16 = non-GM Coker-312 (P1), 17 = Bollgard-II, 18 = negative control (water) * NIBGE-1601 cotton event specific primers: DESPF1 and DESPR1 (fragment size = 239 bp), DESPF1 and DESPR2 (fragment size = 472 bp), DESPF1 and DESPR3 (fragment size = 644 bp).

Three genome walking libraries of NIBGE-1601 cotton were constructed using DraI, EcoRV and SspI restriction enzymes. One of the library (constructed using EcoRV) gave better results and three differentially amplified fragments (double gene event specific fragments; DE1, DE2, DE3) were obtained in its secondary PCR. DE1, DE2 and DE3 fragments were cloned and then sequenced. Nucleotide sequences of DE1, DE2 and DE3 event specific fragments were similar, indicating that they represented the same insertion event. On one end, it matched with the sequence of the double gene construct while its other end was found in cotton genome (*G. hirsutum*) sequence. General and specific BLAST searches at NCBI and CottonGen confirmed sequence similarity of the flanking region to the cotton genome and verified the integration of NIBGE double gene construct into the cotton genome. Based on BLAST search and sequence analysis, NIBGE-1601 event specific primers were designed from the junction region of cotton genome and double gene construct (Table 1, Fig. 4 and Supplementary Fig. S4). NIBGE-1601 event specific primers were evaluated to differentiate NIBGE-1601 event from Monsanto cotton events. NIBGE event specific primers (DESPF1 in combination with DESPR1, DESPR2 and DESPR3) successfully amplified PCR products only from NIBGE-1601 cotton plants. These amplified PCR products were further confirmed successfully through sequencing. The sequencing results showed the 100 percent sequence identity to our expected sequence of event specific region. NIBGE-1601 event specific primers were also used for PCR of genomic DNA of Monsanto events (MON531, MON 1445 and MON 15,985) and other cotton lines including non-GM coker-312 and triple gene NIBGE cotton (event E2) but there was no amplification from these cotton lines (Table 2, Figs. 5, 6 and Supplementary Fig. S5; IPO, Pakistan Patent Application No. 250/2019).

**Table 1:** Primer sequences used for transgene analysis and event specific PCR.

S. no	Primer		Primer Sequence	Product size (bp)	Reference
1	NIBGE Cry1Ac specific	CR1BDR5	ATGTCCATAAGGTGAGGTG	521	^15^
2		CR1BDF5	TTGCGTGAAGAGATGAGG		
3	NIBGE Cry2Ab specific	CR2BDR4	ACTTGAGTGGCGTGTATG	614	
4		CR2BDF4	CGGTGCTAACTTGTATGC		
5	Cotton genome specific	DESPF1	GTCGTATGACTATGTTTAATTTGG	239	This study
6	NIBGE1601 event specific	DESPR1	GAGTGGCTCCTTCAACGTTG		
7	NIBGE1601 event specific	DESPR2	GGCGGAAATAGGTAAAGAAG	472*	
8	NIBGE1601 event specific	DESPR3	TCGTCCTGCAGTTCATTCAG	644*	

*Using DESPF1 as a forward primer.

**Table 2:** Transgene Analysis of Insect Resistant NIBGE-1601 Cotton Event.

Samples & Tests	PCR using specific primers (DNA based test)								
	SadI	nptII	NIBGE Cry1Ac	NIBGE Cry2Ab	NIBGE EPSPS	NIBGE-1601 event specific primers	Mon 531 (BG)	Mon 15,985 (BGII)	Mon 1445 (RR)
NIBGE-1601 cotton	** + **	** + **	** + **	** + **	** − **	** + **	** − **	−	** − **
Coker-312 cotton control (-ve)	** + **	** − **	** − **	** − **	** − **	** − **	** − **	** − **	** − **
BGII + RR MON cotton control	** + **	** + **	** − **	** − **	** − **	** − **	** + **	** + **	** + **

− band absent or no amplification, + band present or amplification.

### NIBGE-1601 event detection method validation

**Figure 7:**
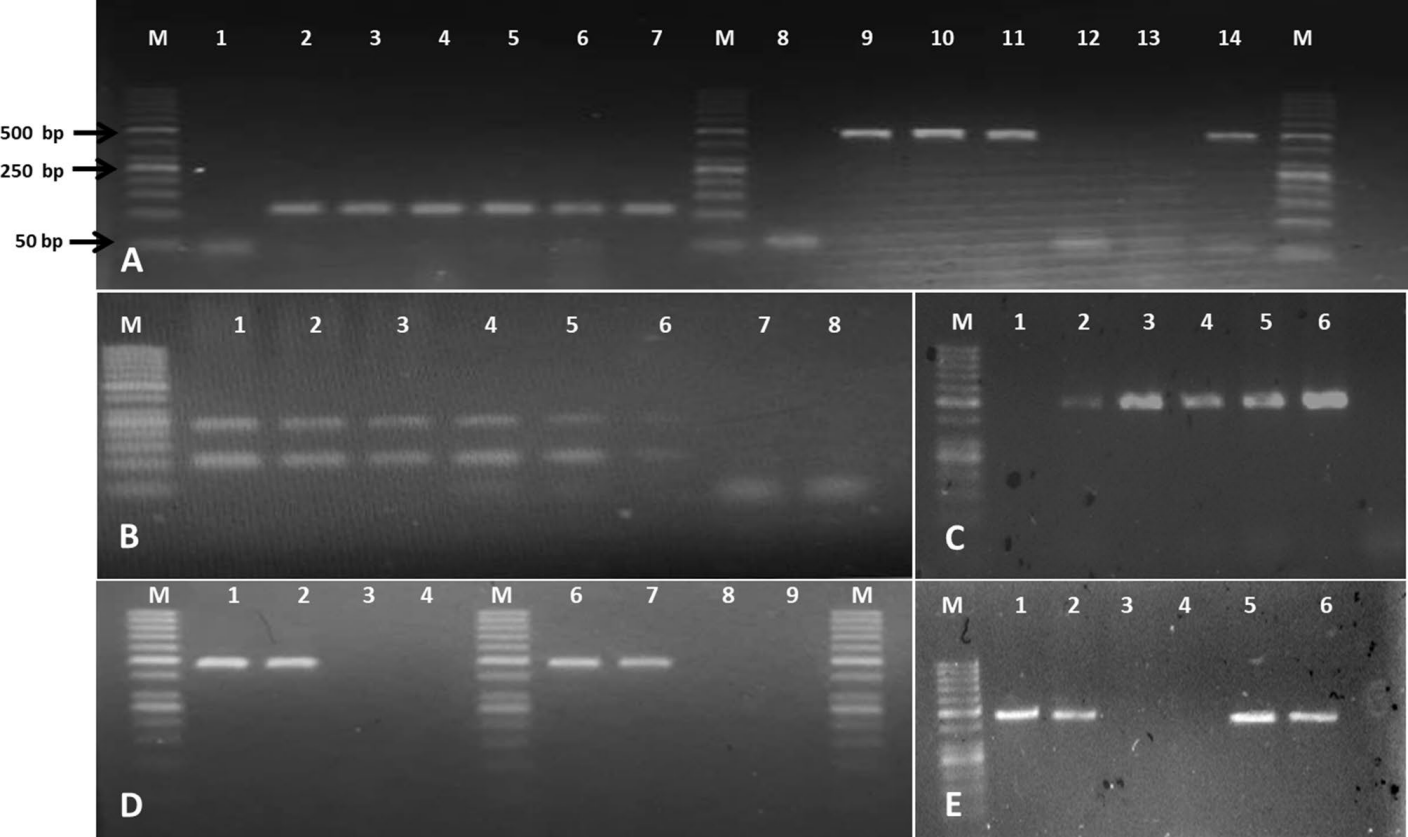
Evaluation of NIBGE-1601 event detection method. (**A**): PCR on Different DNA mixtures for NIBGE-1601 event specific primer specificity. M: 50 bp DNA Marker, Lane 1–7 = SadI amplification (107 bp) and Lane 8–14 = NIBGE-1601 specific amplification (DESPF1/R2; 472 bp) of Samples: Lane 1,8 = Negative water control, Lane 2,9 = NIBGE-1601 & IR-NIBGE 3701 mixture, Lane 3,10 = NIBGE-1601 & non-GM coker-312, Lane 4, 11 = NIBGE-1601 & non-GM rice mixture, Lane 5,12 = Non-GM coker, Lane 6,13 = IR-NIBGE 3701 GM & non-GM coker, and Lane 7,14 = NIBGE-1601 cotton. NIBGE-1601 event specific primer DESPF1&DESPR2 (472 bp) was used for the PCR amplification of DNA mixtures. (**B**) Limit of detection (LOD) for sensitivity of NIBGE-1601 detection. M: 50 bp DNA Marker, Lane 1–7 = Serial dilutions of NIBGE-1601 genomic DNA 100, 50, 10, 1, 0.05 & 0.01 ng, and -ve water control respectively. NIBGE-1601 event specific primer DESPF1&DESPR1 (239 bp) was used for PCR amplification. (**C**) NIBGE-1601 primer efficiency at different MgCl_2_ concentrations. M: 50 bp DNA Marker, Lane 1) Negative water control, Lane 2–6) NIBGE-1601event specific amplification at 0.5 mM, 1 mM, 2 mM, 3 mM and 4 mM concentrations of MgCl_2._ DESPF1&DESPR2 (472 bp) primer pair was used for the PCR using Taq DNA polymerase. (**D**) NIBGE-1601 primer efficiency at different primer concentrations using Taq DNA polymerase. M: 50 bp DNA Marker. Lane 1–4 = Primer concentration used 0.8 µM, Lane 6–9 = Primer concentration used 0.5 µM, Lane 1,2,6,7 = NIBGE-1601 cotton, Lane 3,4,8,9 = Negative water controls, DESPF1&DESPR2 (472 bp) primer pair was used for the PCR using Taq DNA polymerase. (**E**) NIBGE-1601 primer efficiency with different PCR mastermix brands. M: 50 bp DNA Marker. Lane 1,2 = NIBGE-1601 cotton DNA PCR using DreamTaq Green PCR Master Mix, Lane 3,4 = Negative water controls, Lane 5,6 = NIBGE-1601 cotton DNA PCR using 2X PCR Taq Plus MasterMix with dye, DESPF1&DESPR2 (472 bp) primer pair was used for the PCR using Taq DNA polymerase.

NIBGE-1601 event specific primers are highly specific as these give sharp single bands on PCR. It was strengthened by blast analyses that did not discover any off-target hits for these primers. It authenticated the specificity of NIBGE-1601 primers (Supplementary Table S1 and Supplementary Fig. S7). The PCR of NIBGE-1601 cotton DNA mixtures with other plant DNA amplified specific fragment of 472 bp for NIBGE-1601 event, only from mixtures representing NIBGE-1601 DNA, displaying the primer specificity. Moreover, the DNA mixtures without NIBGE-1601 did not amplify any fragment (Fig. 7A). Based on these results we conclude that NIBGE-1601 event specific primers are highly specific for detection of NIBGE-1601 event even from different DNA-mixtures.

PCR amplification on serial dilutions of NIBGE-1601 DNA was detected up to 0.05 ng DNA dilution, however, the trend of amplification decreased from 100 ng to 0.05 ng DNA dilutions. We detected 0.05 ng as the lowest dilution for NIBGE-1601 genomic DNA, where the amplification could be seen on agarose gel (Fig. 7B). Amplification was found on all tested MgCl_2_ concentrations of 0.5 mM, 1 mM, 2 mM, 3 mM and 4 mM, with an increasing trend of amplification from 0.5 to 4 mM MgCl_2_ concentration. The maximum fragment intensity was detected at 4 mM MgCl_2_ (Fig. 7C). The primer pair also efficiently amplified NIBGE-1601 specific event fragment of 472 bp at 0.8 µM and 0.5 µM primer concentrations, where amplifications were sharper with 0.8 µM primer concentration (Fig. 7D). Specific amplification of 472 bp with NIBGE-1601 event specific primer was found for all tested brands of PCR mastermix (Fig. 7E,C). Positive plants of T_1_ and T_2_ progenies of NIBGE-1601 were also tested using single and multiplexing PCRs; the results were similar to that of its T_0_ parent (Supplementary Fig. S6A and S6B). These results indicate that NIBGE-1601 event specific primers are highly specific and can detect NIBGE-1601 event at different PCR conditions using single or multiplexing method.

## Discussion

PCR-based detection of transgenic plants has been proven to be an efficient and reliable method. It is basically due to the stable nature of DNA and sensitivity of PCR technology, which is widely used for transgene and event identification. To differentiate non-GM from GM plants, from a low to high level of specificity, PCR assays are divided into four categories: (i) screening PCR, for widely used regulatory genetic elements like 35S promoter and nos terminator; (ii) transgene specific PCR, for the detection of exogenous genes; (iii) construct specific PCR, for amplification of junction regions of regulatory elements and transgene within the construct like promoter and gene, or terminator and gene specific amplification; iv) event specific PCR with high specificity targets T-DNA and its adjacent sequences of the plant genome^17^. PCR amplification from NIBGE-1601 cotton using gene specific primers of NIBGE Cry1Ac and NIBGE Cry2Ab verified that these genes have sufficient nucleotide variations to be discriminated from Monsanto versions of these two genes. No amplification of Monsanto events from NIBGE-1601 clearly delineated that the NIBGE-1601 cotton event is free from any of the Monsanto events.

A prerequisite for a successful regulatory framework and commercial approval process, is the biosafety evaluation of transgenic events, with molecular characterization as an important component^18^. Identification of unknown DNA sequences flanking known sequence in the genome is very crucial for transgenic event characterization. To determine flanking sequences in the genome, there are different methods like inverse PCR^19^, genome walking^20^ and TAIL-PCR^21^. Genome walking approaches have been continuously modified and improved and more recently coupled with NGS technologies^13,20^. Genome walking protocols are mainly comprised of PCR-based methodologies to identify unknown nucleotide sequences flanking the already known locations in the genome^22–24^. Specific primers are used along with a special type of adaptors that has made genome walking a technology of choice^25–28^.

In the present study, genome walking technology has been successfully employed to characterize the NIBGE-1601 cotton event. Adaptors, primers and restriction enzymes were carefully designed and selected for construction of cotton genome walking libraries, because success of genome walking relies on the presence of specific restriction sites, ligation of adaptor sequence and amplification with specific primers^29^. Event specific PCR is of particular importance for the detection of different lines of transgenic crops in order to meet the regulatory requirements. The proposed event detection method is qualitative in nature and it is limited to identify presence or absence of NIBGE-1601 event, however, its quantitative assay can also be developed and applied to for labeling of GMOs as per international thresholds. NIBGE-1601 event specific primers have high specificity and reliability to clearly differentiate NIBGE-1601 from Monsanto and other cotton lines like non-GM coker-312 and triple gene NIBGE cotton. For better control of bollworms and armyworm, the NIBGE-1601 cotton event harboring Cry1Ac-Cry2Ab can be used as a novel germplasm to develop insect resistant cotton varieties. The NIBGE-1601 event detection method will help cotton breeders because it can be applied to identify desirable plants having NIBGE-1601 blood in different generations during varietal development program. Furthermore, this event detection method would be helpful in the commercialization of NIBGE-1601 cotton, as stakeholders including cotton breeders, seed companies, farmers and regulators will be able to identify the NIBGE-1601 event and seed purity during different varietal development trials, variety approval process and protection of IPRs.

## Materials and methods

### Plant material

Leaves from the NIBGE-1601 cotton plants having double gene (Cry1Ac + Cry2Ab) construct were used for the isolation of high quality genomic DNA. CTAB method^30^ was used for DNA extraction from cotton. Similarly, DNA was isolated from leaves of non-GM coker-312, triple gene NIBGE cotton (E2 event) and MON531 cotton (IR-NIBGE-3701), while genomic DNA of MON1445 and MON15985 cotton was obtained from another experiment.

### PCR analysis

The concentrations of the genomic DNA of non-GM coker-312, triple gene NIBGE cotton (E2 event), MON531 cotton (IR-NIBGE-3701), MON1445 and MON15985 cotton were analyzed using the Nanodrop. NIBGE Cry1Ac and Cry2Ab gene specific primers were used for the PCR amplification. Monsanto event (Mon531 and Mon15985) specific primers were used to check the presence or absence of Monsanto events in NIBGE-1601. Reaction mixture was prepared using Dream Taq Green Master Mix (Thermo Fisher Scientific, Cat No. K1081), 1 µL (10 µM) of each gene specific primer (Table 1), 5 µL (50 ng/ µL) genomic DNA from cotton, and deionized PCR water to make total reaction volume of 25 µL. A Bio-Rad PCR machine (BIO-RAD C1000 96 well PCR Thermal Cycler) was used with PCR profile was set as initial denaturation at 95˚C for 5 min; then 40 cycles with denaturation for 1 min at 94˚C, 1 min for annealing at 55˚C, 1 min extension at 72˚C; and then 10 min final extension at 72˚C. Reagents control and water controls were used as negative controls in PCR. Specific primer sequences for amplification of Sad1, nptII, MON531, MON15985, MON1445 were from the GMO Detection Method Database (GMDD access date July 9, 2020; http://gmdd.sjtu.edu.cn) and already available literature^17,31^. Cotton fiber specific Sad1 primers were used as an internal quality control to check either cotton DNA was amplifiable or not. Gel electrophoresis was performed using 2% agarose gel in 0.5X TAE buffer and run on 8 V/cm, stained with ethidium bromide (10 mg/ ml) and viewed with Gel Doc EZ Imager, Bio-Rad.

### Event characterization of NIBGE-1601 cotton

**Figure 8:**
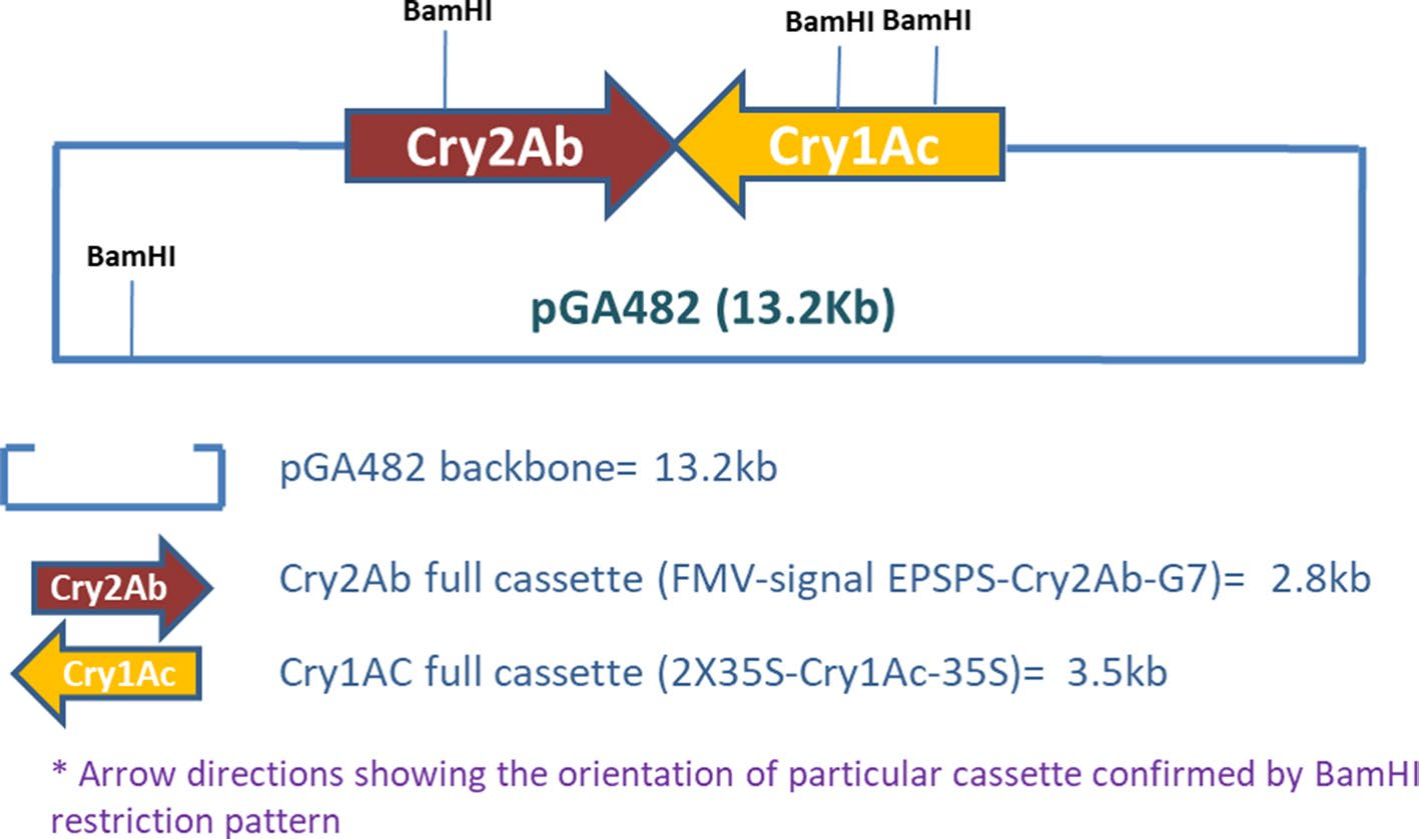
Map of Cry1Ac-Cry2Ab-pGA482 used to develop NIBGE-1601 double gene cotton.

The NIBGE-1601 cotton event was characterized using genome walking technology. Highly purified good quality genomic DNA of confirmed NIBGE-1601 Coker 312 cotton plant was isolated. Three restriction enzymes (DraI, EcoRV and SspI) were selected for complete digestion of the genomic DNA. Digestion was done overnight at 37 °C. Following the strategy of the GenomeWalker Universal Kit (Cat No. 636405, Clontech), specific genome walking adaptors were designed and ligated to restricted genomic DNA for construction of genome walking “libraries”. Then primary PCR and secondary (nested) PCR were run using adaptor specific and gene specific primers. These primers were designed based on double gene construct map (Fig. 8). Long PCR Enzyme mix (Cat No. K0182, Thermo Fisher Scientific) was used for PCR amplifications. Differentially amplified products were cloned and Sanger sequencing was done through a commercial company (Macrogen Inc.) to sequence the cloned PCR products. Based on BLAST search and sequence analysis, NIBGE-1601 event specific primers (Table 1) were designed from the junction region of the cotton genome and double gene construct. Primers were designed using the CLC Main Workbench software. NIBGE-1601 event specific primers were thoroughly evaluated and confirmed to be different from Monsanto cotton events.

### Event detection method validation

Specificity of all NIBGE-event specific primers was accessed by running primer-blast with default parameters using non-redundant (nr), Plants and *Gossypium* specific databases. For event detection method validation, NIBGE-1601 genomic DNA was mixed in different plant DNAs at similar concentration of 50 ng/µl for NIBGE-1601 event specific fragment detection. Different plant DNA used to make DNA mixtures included, GM cotton IR-3701 (Mon531), non-GM coker-312 and non-GM rice. After preparing mixtures of NIBGE-1601 DNA with DNA of other plants, PCR was run on these mixtures using NIBGE-1601 event specific primers DESPF1 & DESPR2. To check the limit of detection, serial dilutions of NIBGE-1601 genomic DNA were prepared as 100, 50, 10, 1, 0.1, 0.05 and 0.01 ng and subjected to qualitative PCR using NIBGE-1601 event specific primers DESPF1 and DESPR1. Moreover, to access NIBGE-1601 specific primers amplification efficiency, PCR was run on NIBGE-1601 cotton DNA using DESPF1 & DESPR2 primers at different concentrations of MgCl_2_ (0.5 mM, 1 mM, 2 mM, 3 mM and 4 mM), at different primer concentrations (0.8 µM and 0.5 µM) and using different PCR mastermix. We used Taq DNA polymerase (Thermo Scientific Cat No. EP0405), DreamTaq Green PCR Master Mix (2X) (Thermo Scientific Cat No. K1082) and 2X PCR Taq Plus MasterMix with dye (Abm Cat No. G901-dye) PCR master mixtures in this experiment. Furthermore, positive plants of T_1_ and T_2_ progenies of NIBGE-1601 were also tested using single and multiplexing methods.

## Supplementary Information

Supplementary Information.
