# Supplementary material for: Development of event-specific detection method for identification of insect resistant NIBGE-1601 cotton harboring double gene Cry1Ac-Cry2Ab construct
Source: Sci Rep. 2021 Feb 10;11:3479. doi: 10.1038/s41598-021-82798-w (PMC7876094; doi:10.1038/s41598-021-82798-w)
Supplement: Supplementary file 1 — Supplementary Information. [file 41598_2021_82798_MOESM1_ESM.pdf]

Development of event-specific detection method for identification of insect resistant NIBGE-1601 cotton harboring double gene Cry1Ac-Cry2Ab construct

Muhammad Asif[1], Hamid Anees Siddiqui[1], Rubab Zahra Naqvi[1], Imran Amin[1], Shaheen Asad[1], Zahid Mukhtar[1], Aftab Bashir[2] & Shahid Mansoor[1]

[1]National Institute for Biotechnology and Genetic Engineering, A Constituent College of Pakistan Institute of Engineering and Applied Sciences, Faisalabad, Pakistan.

[2]Department of Biological Sciences, Forman Christian College, Lahore, Pakistan

***Correspondence:**
Shahid Mansoor
shahidmansoor7@gmail.com

Fig. S1 **PCR of NIBGE cotton using Sad1, nptII and NIBGE Cry1Ac primers**

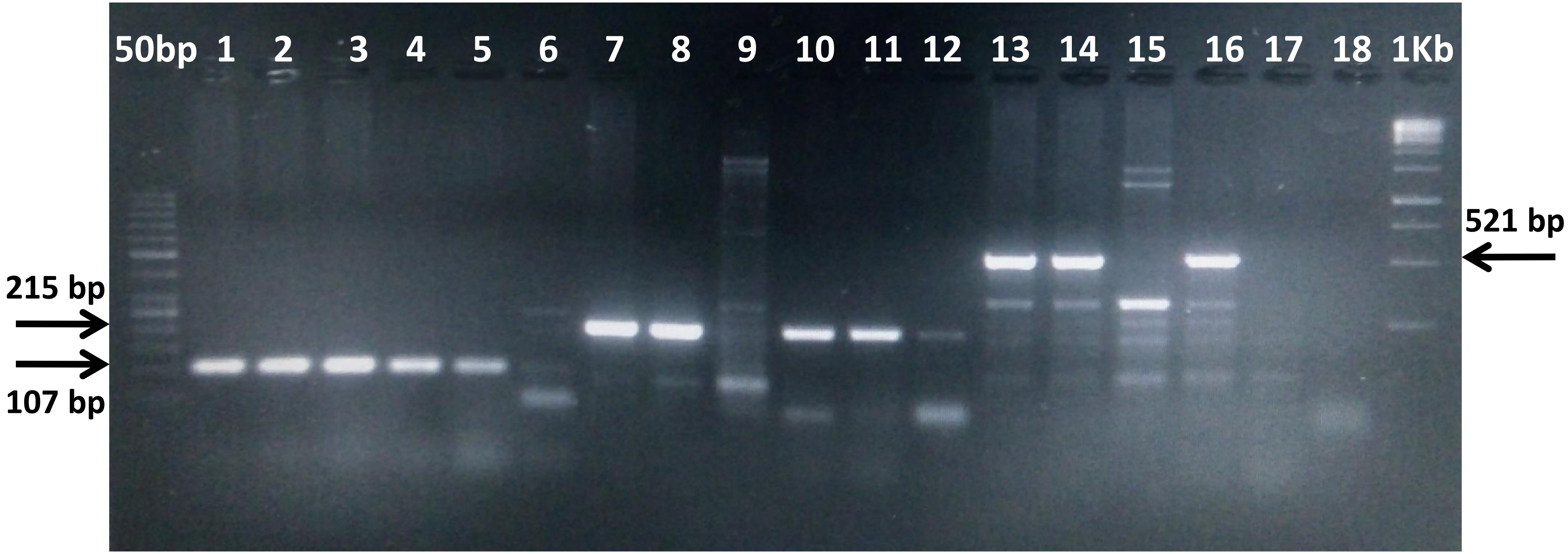

PCR for transgene analysis of NIBGE-1601 cotton event.

1= NIBGE-1601 cotton, 2= triple gene cotton, 3= non-GM Coker (P2), 4= triple gene cotton, 5= Bollgard, 6= negative control (water), 7= NIBGE-1601 cotton, 8= triple gene cotton, 9= non-GM Coker (P2), 10= triple gene cotton, 11= Bollgard, 12= negative control (water), 13= NIBGE-1601 cotton, 14= triple gene cotton, 15= non-GM Coker (P2), 16= triple gene cotton, 17= Bollgard, 18= negative control (water)

*** Primers: S1F+S2R (Sad1; cotton fiber gene specific, 107 bp), APH2 Short+ APH2 Reverse (nptII, 215 bp), CR1BDF5+CR1BDR5 (NIBGE Cry1Ac gene specific, 521 bp)**

Fig. S2 **PCR of NIBGE cotton using NIBGE Cry2Ab, EPSPS and MON531 primers**

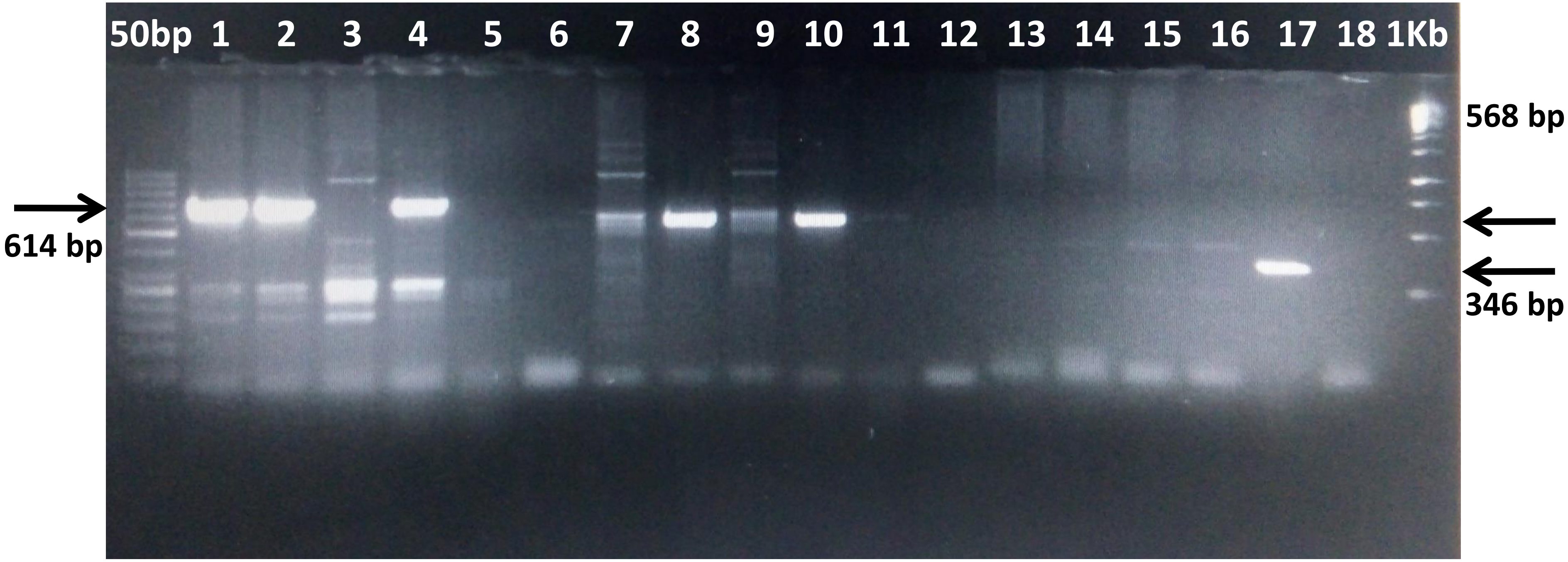

614 bp

568 bp

346 bp

PCR for transgene analysis of NIBGE-1601 cotton event.

1= NIBGE-1601 cotton, 2= triple gene cotton, 3= non-GM Coker (P2), 4= triple gene cotton, 5= Bollgard, 6= negative control (water), 7= NIBGE-1601 cotton, 8= triple gene cotton, 9= non-GM Coker (P2), 10= triple gene cotton, 11= Bollgard, 12= negative control (water), 13= NIBGE-1601 cotton, 14= triple gene cotton, 15= non-GM Coker (P2), 16= triple gene cotton, 17= Bollgard, 18= negative control (water)

*** Primers: CR2BDF4+CR2BDR4 (NIBGE Cry2Ab gene specific,614 bp), EPSF3+EPSR3 (NIBGE EPSPS gene specific, 568 bp), C1F+C2R (MON531, BG event specific, 346 bp)**

Fig. S3

PCR of NIBGE cotton using BGII (MON15985) specific primers

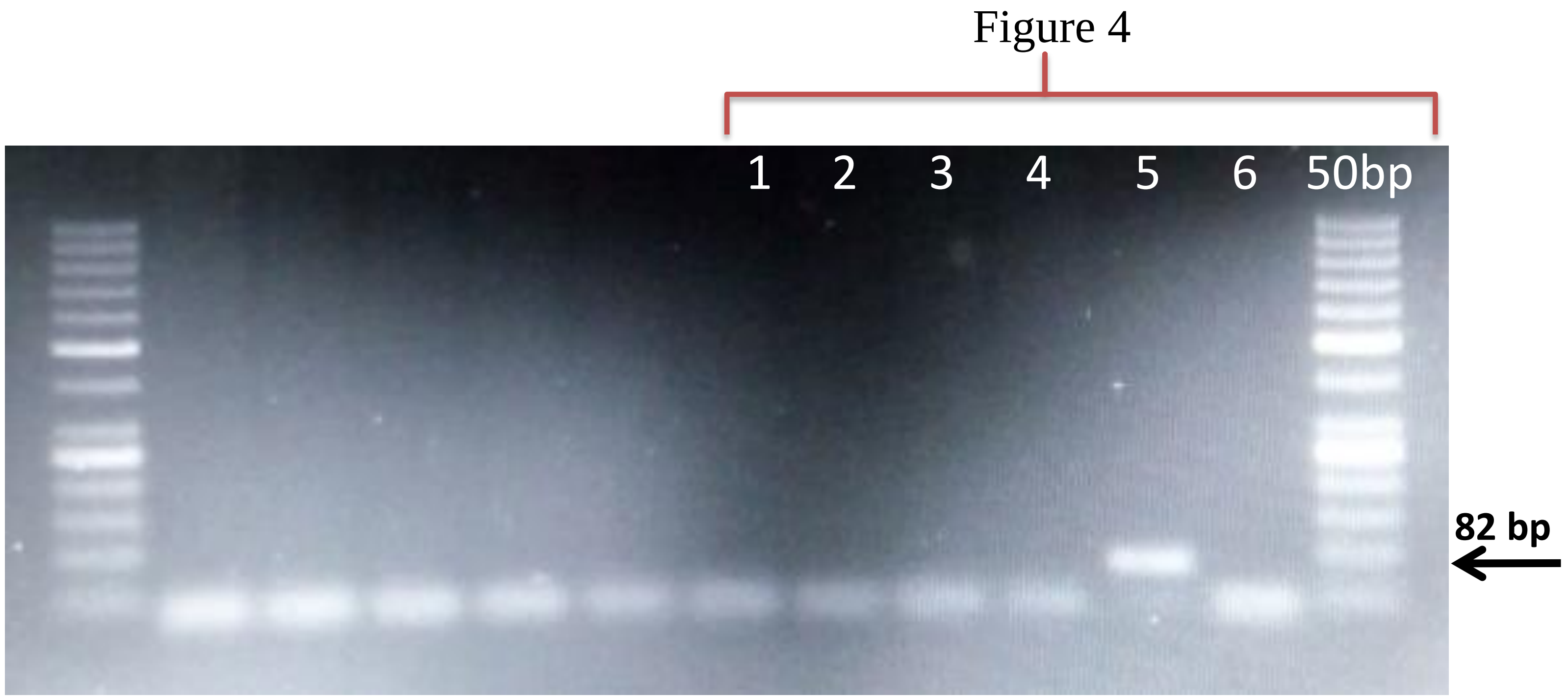

PCR for transgene analysis of NIBGE-1601 cotton event.

<u>1, 2 = NIBGE-1601 cotton plants</u>; 3, 4 = non-GM Coker (P1, P2), 5 = Bollgard-II, 6= negative control (water)

*** Primers: 13_109F and 13_109R (Mon15985, BGII event specific, 82 bp)**

<u>Note:</u>

The relevant portion of the gel image for figure 4 in manuscript was taken from the above gel. The portion taken from this gel for using in manuscript is shown under the brace and is labelled from 1-6 and then 50bp DNA marker.

Fig. S4

PCR for transgene analysis of NIBGE-1601 cotton event using BGII (MON15985) and RR (MON1445) specific primers

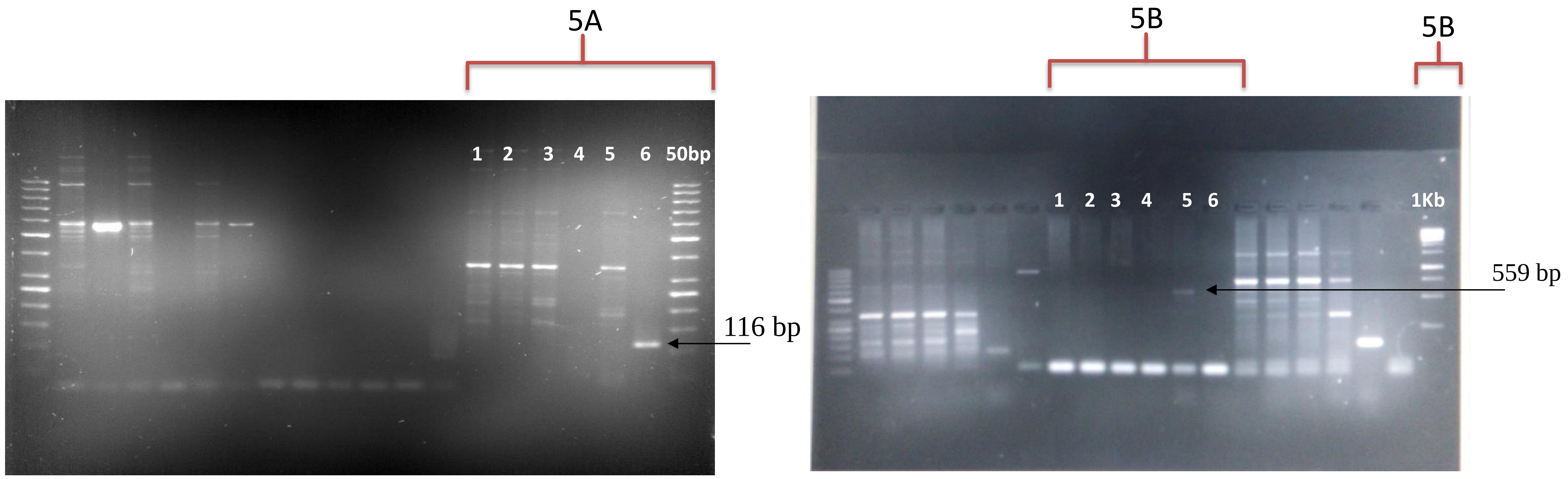

Panel 5A): 1= NIBGE-1601 cotton, 2= triple gene cotton, 3= non-GM coker (P2), 4= negative control (water), 5= non-GM cotton, 6= Bollgard-II

Panel 5B): 1= NIBGE-1601 cotton, 2= triple gene cotton, 3= non-GM Coker (P2), 4= triple gene cotton, 5= RR cotton, 6= negative control (water)

*** Primers: 5A) CTCR2F+CTCR2R (Mon15985, BGII event specific, 116 bp)**
5B) E1F+E2R (Mon1445, RR event specific, 559 bp)

Note:

Figure 5: The relevant portions for gel image for figure 5 (A & B) in the manuscript were taken from these gels. The portions of the gel used in the manuscript with the name of 5A and 5B is shown under the braces.

Fig. S5

PCR of NIBGE and Monsanto cotton using NIBGE-1601 event specific primers

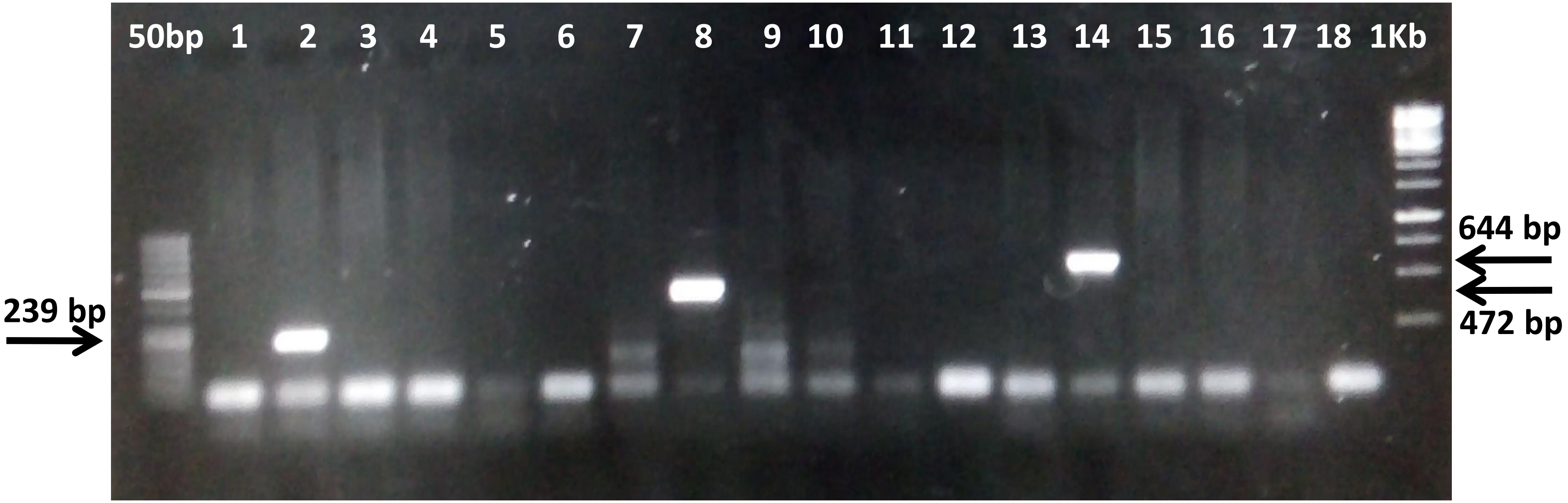

Event specific PCR of NIBGE-1601 insect resistant cotton.

1= GM cotton, <u>2= NIBGE-1601 cotton</u>, 3= triple gene cotton, 4= non-GM Coker (C-P1), 5= Bollgard-II, 6= negative control (water), 7= GM cotton, <u>8= NIBGE-1601 cotton</u>, 9= triple gene cotton, 10= non-GM Coker (C-P1), 11= Bollgard-II, 12= negative control (water), 13= GM cotton, <u>14= NIBGE-1601 cotton</u>, 15= triple gene cotton, 16= non-GM Coker (C-P1), 17= Bollgard-II, 18= negative control (water)

*** NIBGE-1601 cotton event specific primers:**

DESPF1 and DESPR1 (fragment size= 239 bp), DESPF1 and DESPR2 (fragment size= 472 bp), DESPF1 and DESPR3 (fragment size= 644 bp)

Fig. S6-A

NIBGE-1601 event specific PCR T1 and T2 generation

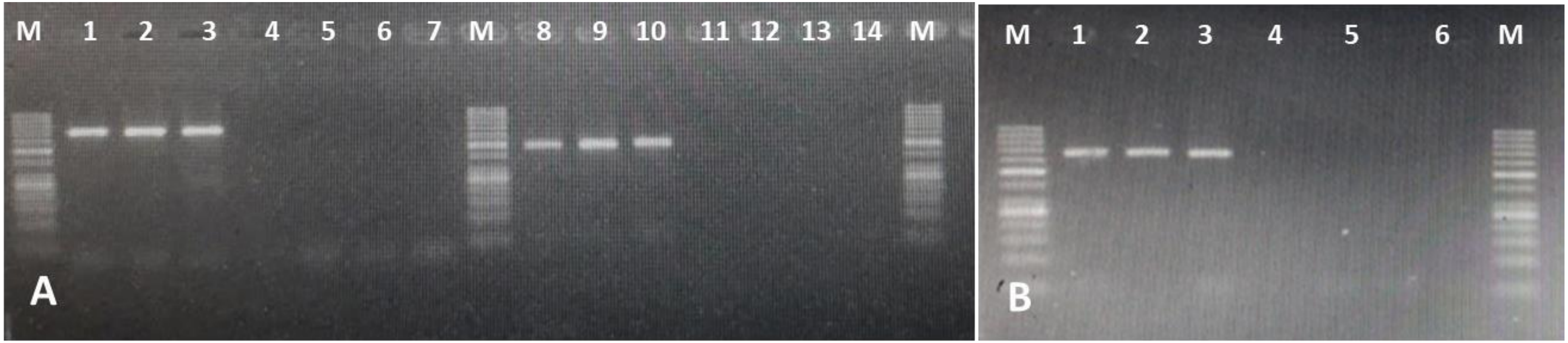

Singleplex PCR on T1 and T2 plants of NIBGE-1601 using NIBGE gene specific primers.

Panel A. M: 50bp DNA Marker. PCR with Cry2Ab primers: Lane 1,2= NIBGE-1601 cotton DNA T1 plants, Lane 3= NIBGE-1601 cotton DNA T2 plant, showing amplification of Cry2Ab specific fragment (614 bp), Lane 4,5= Non-GM coker-312, Lane 6,7= Negative water controls. PCR with Cry1Ac primers: Lane 8,9= NIBGE-1601 cotton DNA T1 plants, Lane10= NIBGE-1601 cotton DNA T2 plant, showing amplification of Cry1Ac specific fragment (521 bp), Lane 11,12= Non-GM coker-312, Lane 13,14= Negative water controls.

Panel B. M: 50bp DNA Marker. PCR with DESPF1&DESPR3 NIBGE event specific primers: Lane 1,2= NIBGE1601 cotton DNA T1 plants, Lane 3= NIBGE-1601 cotton DNA T2 plant, showing amplification of NIBGE-1601 specific fragment (644 bp), Lane 4,5= Non-GM coker-312, Lane 6,7= Negative water controls.

Fig. S6-B

NIBGE-1601 event specific PCR T1 and T2 generation

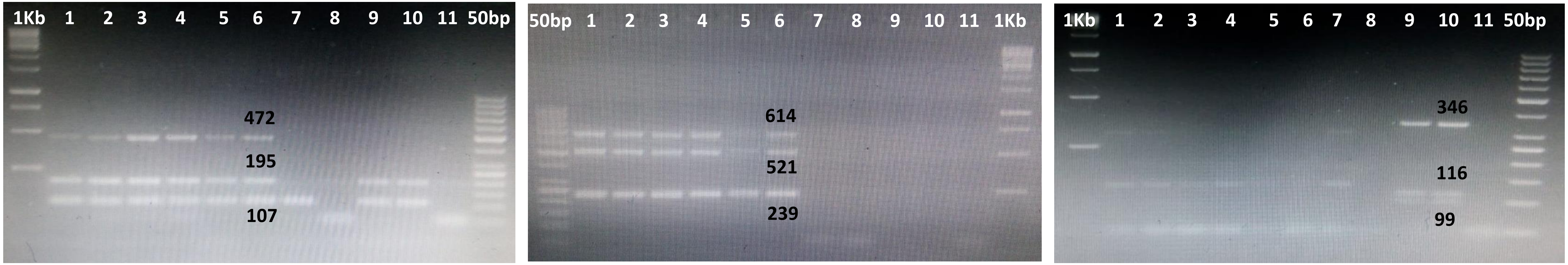

Mutiplex PCR for transgene analysis and event detection of NIBGE-1601 cotton:

1kb and 50bp= DNA ladders, 1 to 5= NIBGE-1601 T2 plants, 6= NIBGE-1601 T1 plant, 7= Non-GM coker 312, 8= Reagent control (negative), 9 & 10= BG+BGII+RR (Monsanto cotton mix DNA control), 11= PCR water control (negative control)

Panel A: 107 bp= Sad1 cotton fiber gene specific product (S1F & S2R primers),
195 bp= 35S promoter specific product (p35SF & p35SR primers),
472 bp= NIBGE-1601 cotton event specific product (DESPF1 & DESPR2 primers)

Panel B: 239 bp= NIBGE-1601 cotton event specific product (DESPF1 & DESPR1 primers),
521 bp= NIBGE Cry1Ac gene specific product (CR1BDF5 & CR1BDR5 primers),
614 bp= NIBGE Cry2Ab gene specific product (CR2BDF4 & CR2BDR4 primers)

Panel C: 99 bp= Roundup Ready (RR) Monsanto cotton event Mon1445 specific product (E3F and E4R primers),
116 bp= Bollgard-II (BGII) Monsanto cotton event Mon15985 specific product (CTCR2F & CTCR2R primers),
346 bp= Bollgard (BG) Monsanto cotton event Mon531 specific product (C1F & C2R primers)

Note: Monsanto cotton event specific products were not found in NIBGE-1601 cotton.

Table S 1. Primer blast analysis revealed no off targets for all primer sets

Primer		Primer Sequence	Product size (bp)	Primer Blast Similarity (nr, Plants and Gossypium databases)
**Cotton genome specific**	DESPF1	GTCGTATGACTATGTTTAATTTGG	239	No target templates
**NIBGE1601 event specific**	DESPR1	GAGTGGCTCCTTCAACGTTG		
**NIBGE1601 event specific**	DESPR2	GGCGGAAATAGGTAAAGAAG	472*	No target templates
**NIBGE1601 event specific**	DESPR3	TCGTCCTGCAGTTCATTCAG	644*	No target templates

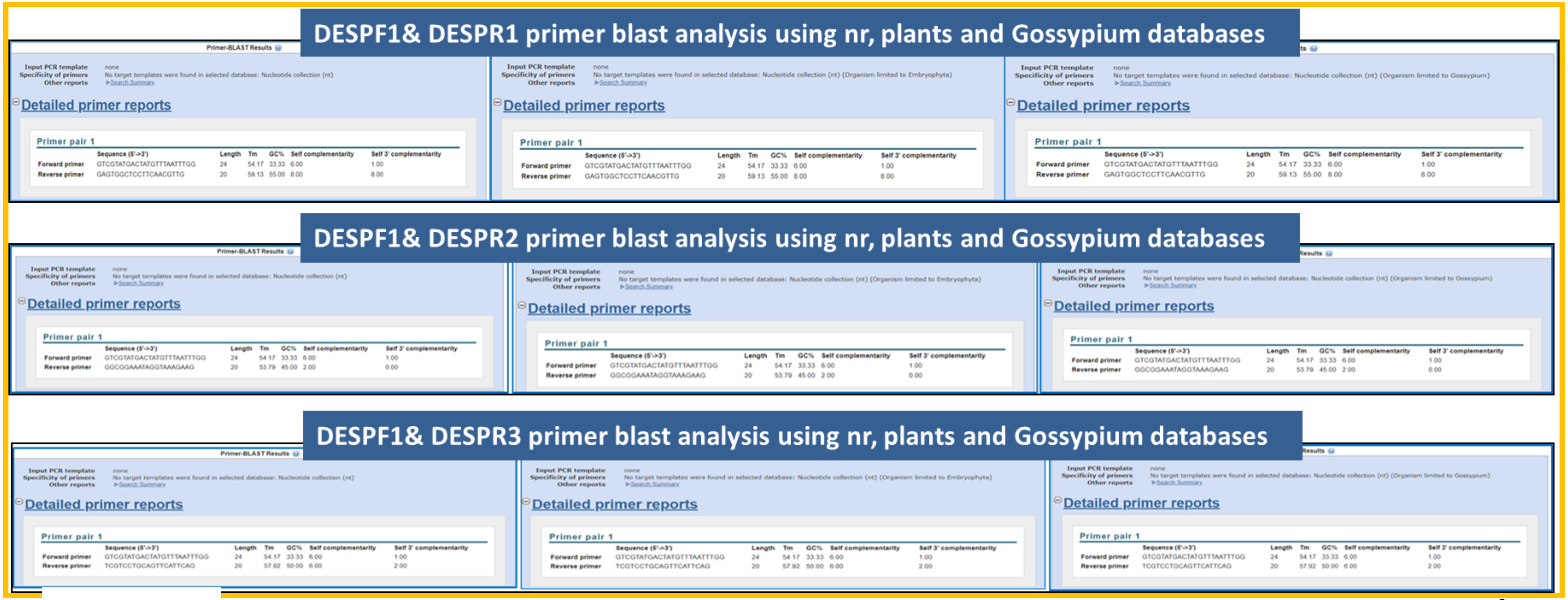

Fig. S7 Insilico primer blast results for NIBGE-1601 event specific primer pairs
